# Comparison of Common Enrichment Broths Used in Diagnostic Laboratories for Shiga Toxin—Producing *Escherichia coli*

**DOI:** 10.3390/microorganisms9030503

**Published:** 2021-02-27

**Authors:** Michael Bording-Jorgensen, Hannah Tyrrell, Colin Lloyd, Linda Chui

**Affiliations:** 1 Department of Laboratory Medicine and Pathology, University of Alberta, Edmonton, AB T6G 2R3, Canada; bordingj@ualberta.ca (M.B.-J.); hstyrrell@gmail.com (H.T.); cdlloyd@ualberta.ca (C.L.); 2 Alberta Precision Laboratories-Public Health Laboratory (ProvLab), Edmonton, AB T6G 2J2, Canada

**Keywords:** STEC, enrichment, real-time PCR, broth, culture

## Abstract

Acute gastroenteritis caused by Shiga toxin-producing *Escherichia coli* (STEC) affects more than 4 million individuals in Canada. Diagnostic laboratories are shifting towards culture-independent diagnostic testing; however, recovery of STEC remains an important aspect of surveillance programs. The objective of this study was to compare common broth media used for the enrichment of STEC. Clinical isolates including O157:H7 as well as non-O157 serotypes were cultured in tryptic soy (TSB), MacConkey (Mac), and Gram-negative (GN) broths and growth was compared using culture on sheep’s blood agar and real-time PCR (qPCR). In addition, a selection of the same isolates was spiked into negative stool and enriched in the same three broths, which were then evaluated using culture on CHROMagar^TM^ STEC agar and qPCR. TSB was found to provide the optimal enrichment for growth of isolates with and without stool. The results from this study suggest that diagnostic laboratories may benefit from enriching STEC samples in TSB as a first line enrichment instead of GN or Mac.

## 1. Introduction

Acute gastroenteritis (AGE), which is defined as vomiting and/or diarrhea for less than 7 days, affects more than 4 million individuals in Canada [1]. Shiga toxin-producing *Escherichia coli* (STEC) are one of the pathogens responsible for major outbreaks. Although their primary reservoir is ruminants, there have been recent outbreaks involving contaminated food items such as flour, clover sprouts, and cheese [2]. STEC infections are associated with hemorrhagic colitis with the possibility of developing hemolytic uremic syndrome [3]. This potentially deadly consequence is due to the production of Shiga toxins (Stx) 1 and/or 2, with Stx 2 having a higher association [4]. The exotoxin genes are located on a lambda prophage and the toxins are released into the lumen during colonization and replication resulted in causing damages to the intestinal barrier [5]. STEC can also contain a variety of virulence factors such as *eae* and *hly,* which are localized within the locus of enterocyte effacement pathogenicity island [6]. 

To date, there have been over 200 serotypes of *E. coli* identified to contain the Shiga toxin and cause diarrheal disease in humans [7]. *E. coli* O157:H7 was the first serotype identified in 1982, causing AGE-related morbidity involved in several outbreaks in the United States of America. Recently, other serotypes known as the “Big 6” (O26, O45, O103, O111, O121, and O145) have been the cause of outbreak in North America [8]. Recent genomic comparisons of O145 have shown significant metabolic diversity within a particular serotype, highlighting the possibility of a difference in growth requirements for enrichment both within and between serotypes, which is required for the identification of serotypes during outbreaks [9]. 

STEC infection is notifiable in Alberta, indicating its importance for monitoring and control. This is achieved through surveillance programs, which rely on the ability to culture the organism for further characterization. Although culture-independent diagnostic testing (CIDT) has become more prominent in recent years, culture is essential for surveillance and cluster detection. Isolation of O157 STEC in the diagnostic laboratory can be achieved using sorbitol-MacConkey agar or chromogenic agar (O157 and non-O157), which are both selective media, but they might not support growth of all serotypes [10]. The Centers for Disease Control and Prevention published guidelines for the diagnosis of STEC in which they recommend either MacConkey or Gram-negative broth for enrichment [11]. Cefixime and tellurite are common ingredients used in the selective agar recommended for the isolation of STEC due to the particularly difficult nature regarding the isolation of non-O157 serotypes [10,12,13]. In contrast, the ingredients of the recommended broth for the enrichment of STEC are less selective and can be used for the growth of multiple pathogens. The objective of this study was to evaluate the growth of clinical isolates with and without stool using various broth media (tryptic soy broth, MacConkey, and Gram-negative) by culture and real-time PCR (qPCR).

## 2. Materials and Methods

### 2.1. Bacterial Isolate Selection and Enrichment

STEC isolates are routinely submitted to the Alberta Precision Laboratories-ProvLab for further characterization. A total of 52 isolates consisting of O157 and non-O157 serotypes as shown in Supplementary Table S1 were included in this study. Archived fingerprinting patterns generated by pulsed-field gel electrophoresis in our database were analysed using BioNumerics software V6.1 (Austin, TX, USA) to ensure that they all have indistinguishable pulsotypes. These isolates were retrieved from skim milk stored at −80 °C and cultured on sheep blood agar plates (BAP) (Oxoid, Fisher Scientific, Ottawa, ON, Canada) overnight at 37 °C. A single colony was picked, suspended in 250 µL of 1× PBS, and 50 µL was added to 5 mL of tryptic soy broth (TSB, Bacto/BD, Fisher Scientific, Ottawa, ON, Canada), MacConkey (MAC, Dalynn Biologicals, Calgary, AB, Canada) and Gram-negative (GN, Dalynn Biologicals) broths, and incubated overnight at 37 °C. A 10-fold serial dilution of each broth were then plated in triplicates onto BAP and colonies were counted the following day.

### 2.2. Spiking of Negative Stool

Negative stools (*n* = 3) were screened for the presence of *stx*_1_ and *stx*_2_ genes using qPCR as well as plated on CHROMagar^TM^ STEC (Dalynn Biologicals, Calgary, AB, Canada) plates to ensure there were no other bacteria within the stool that would grow mauve-colored colonies [14], which might be indicative of STEC colonies. These negative stools were then pooled together for the spiking experiments. A subset of STEC isolates (*n* = 25), which are indicated in Supplementary Table S1, from the broth experiments described above were grown in TSB overnight. Then, 1 mL of a 0.5 O.D. was centrifuged (13,000× *g* for 10 min) and washed with 1× PBS twice. Dilutions were made to obtain a cell suspension of 1 × 10^5^ CFU/mL, and 100 μL was combined with 150 μL of negative stool and added to three separate 5 mL broth tubes (TSB, Mac, GN). The stool-spiked broth cultures were incubated overnight at 37 °C. Ten-fold serial dilutions of each broth were then plated in triplicates on CHROMagar^TM^ STEC (Dalynn Biologicals) and colonies were counted the following day.

Production of the Shiga toxin was determined using Shiga Toxin Quik Chek^TM^ (TechLab, Blacksburg, VA, USA) as per manufacturer’s protocol. In brief, 100 μL of broth was added to a tube containing 650 μL diluent and conjugate, and 500 μL was added to the cassette and left at room temperature for 15 min. Next, 300 μL of wash buffer was added followed by substrate and left to develop at room temperature for 10 min. The results were visually read as either positive or negative for Shiga toxin 1 and 2. The *stx* status of each isolate was already known prior to the experiments; therefore, the Quik Chek^TM^ was used to ensure the growth was due to STEC in the stool experiments (Table S1).

### 2.3. DNA Extraction and qPCR of Broth Enrichment

Enriched TSB, Mac, and GN broths without (*n* = 52) and with (*n* = 25) stool were extracted using rapid lysis buffer (100 mM NaCl, 10 mM Tris-HCL pH 8.3, 1 mM EDTA pH 9.0, 1% Triton X-100). A 250 µL volume of enriched culture was centrifuged (13,000× *g* for 10 min) and the pellet was resuspended in 100 µL of rapid lysis buffer and heated to 95 °C for 15 min using a heating block. The samples were then centrifuged (13,000× *g* for 10 min), and the supernatant was stored at 4 °C until further testing via qPCR.

The primers and probes (Integrated DNA Technology, Coralville, IA, USA) for detecting *stx* genes are shown in Table 1. The total reaction contained 12.5 μL of 1× PrimeTime^®^ Gene Expression Master Mix (Integrated DNA Technology), 0.33 μM of each primer, 0.22 μM probe, 5 μL DNA template and molecular biology grade water in a total of 25 μL reaction volume. A negative template control and O157 positive control DNA was included in each run. qPCR amplification conditions consisted of 95 °C for 1 min followed by 40 cycles of 95 °C for 5 s and 58 °C for 45 s performed on the 7500 FAST real-time PCR system (Applied Biosystems, Foster City, CA, USA). Using a crossing threshold of 0.1, all Ct values below 30 were considered positive.

### 2.4. Statistics

Statistical analysis was performed using Prism8 for Mac (Graph Pad, San Diego, CA, USA). One-way ANOVA with Holm–Sidak’s multiple comparison test were used for statistical comparisons of media growth within each isolate. Bar graphs represent the mean ±SEM and all comparisons with *p* ≤ 0.05 were considered significant.

## 3. Results

### 3.1. Comparing Enrichment of STEC in Different Broths with and without the Presence of Stool

**Figure 1:**
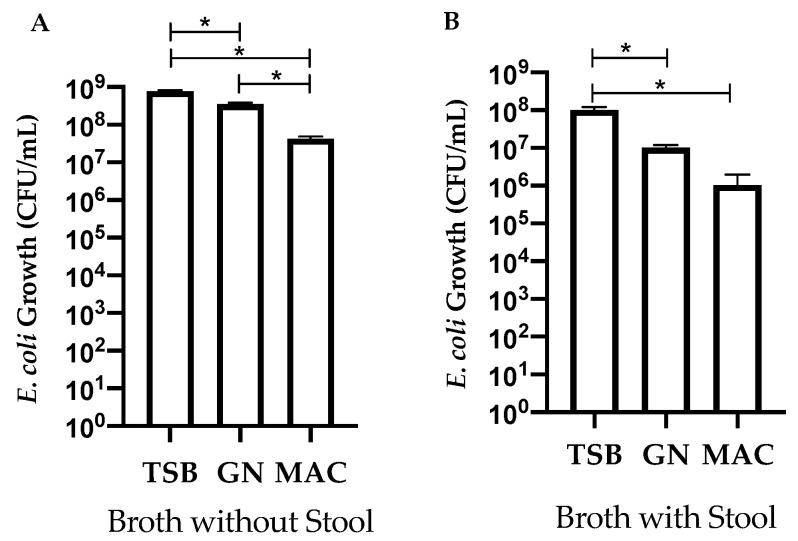
Effect of the tryptic soy broth (TSB), Gram-negative (GN), and MacConkey (MAC) broths for *E. coli* (STEC) enrichment in the absence ((**A**), *n* = 52 isolates) or presence ((**B**), *n* = 25 isolates) of stool. Bars represent the CFU/mL calculated from serial dilutions on blood agar plates (**A**) or CHROMagar^TM^ (**B**). Error bars represent the standard error of the mean. * Indicates growth is significant; ANOVA *p* *≤* 0.05.

STEC isolates (*n* = 52) were grown in TSB, GN, and Mac broth to determine if broth composition would influence growth. Overall, growth in TSB was significantly (*p ≤* 0.5) higher than GN or Mac broth (Figure 1A). GN broth also showed significantly (*p ≤* 0.5) higher growth compared to Mac broth (Figure 1A). A subset of isolates (*n* = 25) was then grown in the same broth with the presence of STEC negative stool. TSB showed significantly higher (*p* *≤* 0.5) growth as compared to GN and Mac in the presence of stool (Figure 1B). There was no significant growth difference between GN and Mac broth (Figure 1B). Growth was overall lower for each of the broths containing stool as compared to no stool; however, there was no statistical difference (Figure 1A,B).

### 3.2. Comparison of Isolates within Each Serotype Group Independent of Stool

**Figure 2:**
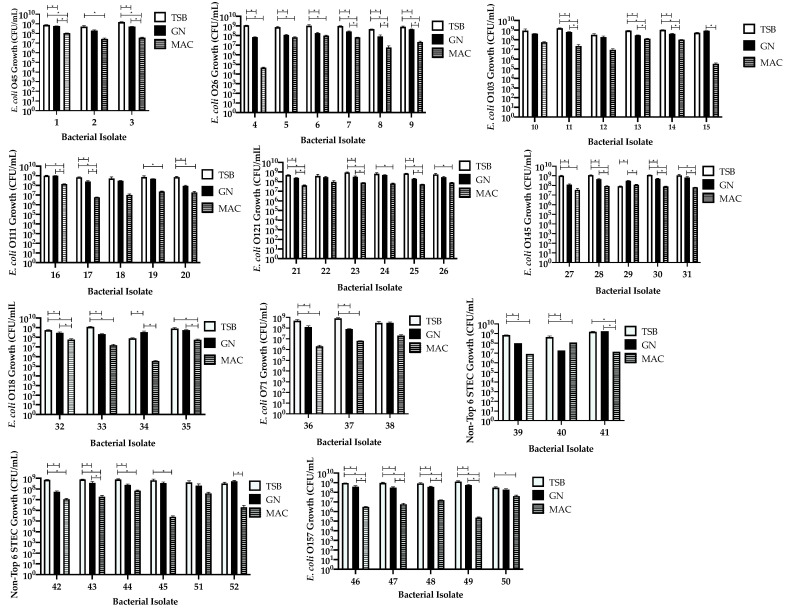
Effects of different enrichments broths tryptic soy broth (TSB), GN, and MacConkey (MAC) on the growth of STEC isolates (*n* = 52 isolates). Bars represent the CFU/mL calculated from serial dilutions plated in triplicate on BAP. Error bars represent the standard error of the mean. * Growth is significant; ANOVA *p* ≤ 0.05.

We compared the growth of all the isolates (*n* = 52) to determine if there was a difference between or within each of the serotype groups when enriched in each of the broth used. STEC were grown on BAP plates and enumerated for comparison between broths. TSB was found to significantly (71%, *p ≤* 0.5) improve growth compared to GN broth for 38 isolates (Figure 2, Table 2). TSB was shown to improve growth significantly (*p ≤* 0.5) as compared to Mac broth for 81% (*n* = 42) of the isolates (Figure 2, Table 2). There was significant growth improvement in GN broth (*p ≤* 0.5) when compared to Mac broth for 28 (54%) isolates (Figure 2, Table 2). Surprisingly, GN showed significant (*p ≤* 0.5) growth as compared to TSB in isolates #29 (O145) and #34 (O118) (Figure 2, Table 2). Growth was found to be decreased in Mac broth for all isolates, although there was no significant difference in broth type for six (12%) isolates (#10 (O103), #12 (O103), #18 (O111), #22 (O121), #38 (O71), and #51 (O5)) (Figure 2).

### 3.3. Comparing Isolate Growth in the Presence of Stool

**Figure 3:**
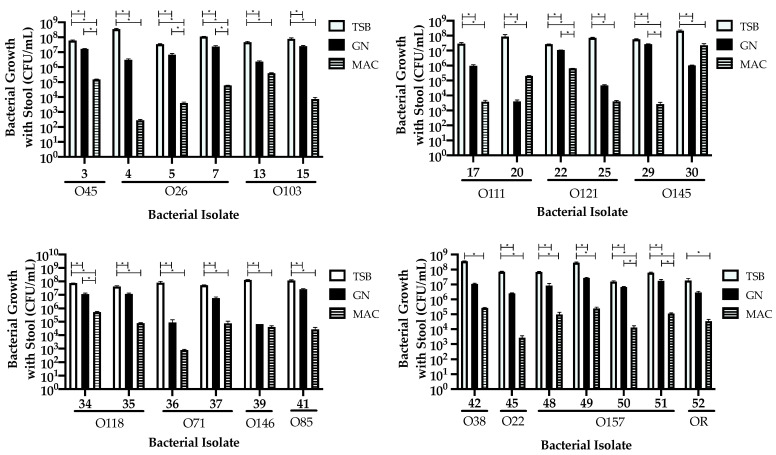
Effects of different enrichments broths tryptic soy broth (TSB), GN, and MacConkey (MAC) on the growth of STEC isolates (*n* = 25 isolates) with stool. The bacterial serotype is indicated. Bars represent the CFU/mL calculated from serial dilution plates in triplicate on CHROMagar^TM^ plates. Error bars represent the standard error of the mean. * Growth is significant; ANOVA *p* *≤* 0.05.

Selected isolates (*n* = 25) from the previous experiment were grown in the same broth type in the presence of stool to determine if their growth would be impacted in the presence of competing bacteria. The stool was first confirmed to be negative for STEC as well as no growth on CHROMagar^TM^ STEC from other bacteria. Blood agar plates were not used for enumeration as they support growth of commensal *E. coli* and other enteric bacteria found in stools. CHROMagar^TM^ STEC was selected for the culture media for performing plate counts. Growth in TSB was found to be significantly (*p ≤* 0.5) higher compared to both GN (23 isolates, 92%) and Mac broths (25 isolates, 100%) (Figure 3, Table 3). GN broth improved growth significantly (*p ≤* 0.5) when compared to Mac broth for isolates #3 (O45), #5 (O26), # 7 (O26), #22 (O121), #29 (O145), #34 (O118), #50 (O157), and #51 (O5), as illustrated in Figure 3.

### 3.4. Comparing Ct Values Targeting the Stx Gene for Isolates Inoculated into Broth with and without Stool

**Figure 4:**
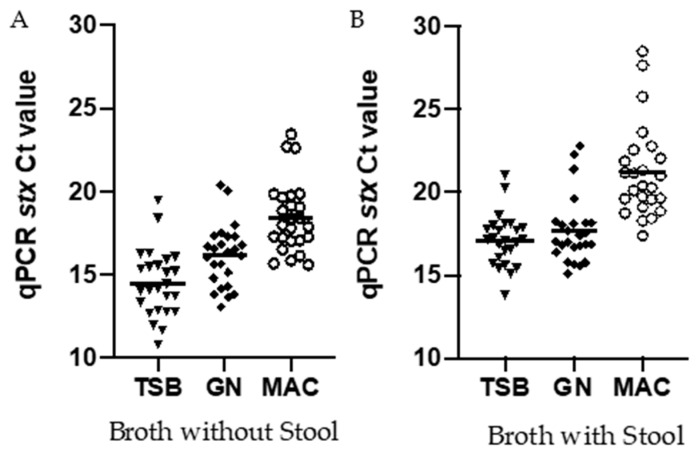
(**A**) represent broth without Stool, (**B**) represent broth with Stool. qPCR Ct values of *stx* from STEC isolates enriched in tryptic soy broth (TSB), GN, and MacConkey (MAC) with and without stool. DNA was extracted from the STEC enriched broth and qPCR was done. Each symbol represents a single isolate. *n* = 25 isolates.

DNA was extracted from the TSB, GN, and Mac broths from both pure cultures and stool-spiked enrichments and the relative abundance of STEC was compared using qPCR crossing threshold (Ct) values. Similar to the observations using colony enumeration, Ct values were consistently lower with DNA extracted from isolates grown in TSB (14.7; 95% CI 13.64–15.31) as compared to GN (16.14; 95% CI 15.38–16.90) or Mac (17.55; 95% CI 17.55–19.32) broths (Figure 4) was observed. The Ct values were higher with DNA from isolates grown in all broth types in the presence of stool; however, the trend remained the same with TSB (17.11; 95% CI 16.46, 17.75), showing lower Ct values as compared to GN (17.67; 95% CI 16.85, 18.49) and Mac (21.18; 95% CI 20.03, 22.33) (Figure 4).

## 4. Discussion

STEC is a major cause of global AGE and is responsible for many notable foodborne outbreaks. Recently, there has been an increased prevalence of serotypes other than O157:H7 implicated in these outbreaks [16]. These associations indicate an urgency for increased surveillance of these serotypes, particularly those deemed the “Big 6” in North America (O26, O45, O103, O111, O121, and O145) [17]. The use of CIDT is becoming more widespread due to its fast turnaround time for reporting, as well as the high sensitivity and specificity. However, culture still remains essential for surveillance purposes, epidemiological investigations, and early cluster detection. Culturing from a stool sample can be particularly challenging due to the presence of the patient’s own microbiome; therefore, it is crucial that the optimal culture media and conditions be applied for the specific pathogen involved. To our knowledge, this is the first study to publish data comparing enrichment broths used in diagnostic laboratories for the culturing of STEC from patient stool samples.

The first aim of this study was to determine whether known STEC isolates would have different growth dynamics with respect to each of the broth tested. GN and Mac broths are selective media commonly used for enteric bacteria such as *E. coli* due to the basic nutrients provided [18,19] as compared with TSB, which is a general medium. *E. coli* is usually regarded as a non-fastidious organism that grows well in most conditions; however, as illustrated in our data, we have found both GN and Mac broths are limited in their ability to support the growth of certain STEC, as expected. Mac broth consistently showed reduced growth as compared to TSB and GN, although there was no indication that this occurred within a particular serotype. Instead, the data suggest that Mac broth may be lacking a particular nutrient as compared to the others, which these isolates require for growth. One such possibility is that Mac broth contains lactose, whereas TSB and GN broth have glucose as the main carbon source. Another speculation is that these isolates might have mutations within the Lac operon which limits their ability to utilize lactose as efficiently as their main carbon source, which is not unique, as other mutations have been found in STEC that affect their metabolic profile [20]. Future study of the biochemical pathways of these organisms will help to understand the growth performance of these isolates in such media. *E. coli* are normally lactose fermenting organisms, which allows for their distinction from *Shigella* spp when diagnosing diarrheal infections. However, there has been evidence that the *stx* lambdoid phage can disrupt the metabolic pathways, particularly in those with *stx*_2a_ [21]. We did not subtype the *stx* gene in these isolates; however, some of the STEC isolates with *stx*_1_ also showed decreased growth in MAC broth, suggesting this may not be limited to *stx*_2a_.

The addition of stool further complicated the growth of many of the isolates we used; however, TSB remained as the most supportive enrichment broth. Stool samples are complex matrices due to the patient’s microbiome, which may affect the growth of STEC. The Enterobacteriaceae family found within the microbiome is of particular importance as they can be easily cultured on MacConkey agar [22]. This competition between microbiome and STEC may be the reason why the growth in Mac broth was significantly lower with the addition of stool compared to enrichment using TSB and GN broth. The addition of stool introduces competition between STEC and organisms present in the microbiome for the limited nutrients included in the broth, which would explain why some of the isolates had considerably lower growth in Mac broth. Isolates #20 and #30 were interesting because there was more growth in Mac broth as compared to GN, suggesting that these isolates could not compete well with the organisms found in the stool when enriched in GN broth as compared to Mac broth, as shown in Figure 3, and such a phenomenon was not observed when this isolate was enriched in the absence of stool (Figure 2).

As CIDT use has increased over the last few years, we performed qPCR to determine if we would see differences between different enrichment broths using a molecular assay. We found that the Ct values reflected the same trend, as was observed by plate enumeration in the absence of stools. Using rapid lysis buffer on the broths containing stool is considered a “crude” method, and there is a chance for PCR inhibition; however, an increase in the Ct values between the different broths corresponded to the decrease in CFU by colony counting. It is most likely that any PCR inhibitors were diluted in the broth during enrichment and therefore did not affect our results. Amplification using DNA from isolates grown in TSB broth showed a mean lower Ct values as compared to both GN and Mac broths. Therefore, if an overnight enrichment is required for the stool samples for the detection of STEC using an enzyme immunoassay, Mac and GN may not be the broths of choice, because they might not provide the optimal growth of STEC. Consequently, depending on the sensitivity of the EIA being used by the diagnostic microbiology laboratory, the toxin level might not be sufficient enough to be detected by the assay. Therefore, it is important that the sample be enriched in the appropriate media to ensure an accurate diagnosis.

## 5. Conclusions

This study highlights the incredible diversity found both within and between STEC serotypes in terms of their enrichment requirements. The enrichment broth chosen by the diagnostic laboratory can greatly influence how well they are able to culture STEC for their detection and further analysis. Based on the results of this study, we suggest that diagnostic laboratories currently using GN or Mac broth may benefit from switching to TSB, which was more supportive of STEC growth. In addition, we hope to alert manufacturers that GN and Mac broth are not optimized for the enrichment of STEC and instead recommend the use of TSB.

## Figures and Tables

**Table 1:** Primer and probe sequences used in this study [15].

Reference Gene, Primer/Probe	Sequence 5′-3′
*stx*_1_-F	TTT GTY ACT GTS ACA GCW GAA GCY TTA CG
*stx*_1_-R	CCC CAG TTC ARW GTR AGR TCM ACR TC
*stx*_1_-P	CTG GAT GAT CTC AGT GGG CGT TCT TAT GTA A
*stx*_2_-F	TTT GTY ACT GTS ACA GCW GAA GCY TTA CG
*stx*_2_-R	CCC CAG TTC ARW GTR AGR TCM ACR TC
*stx*_2_ -P	TCG TCA GGC ACT GTC TGA AAC TGC TCC
In the sequences: Y is (C, T), S is (C, G), W is (A, T), R is (A, G), M is (A, C).	

**Table 2:** Percentage of isolates with significantly different growth in each broth (*n* = 52).

Broth	TSB	GN	Mac
TSB	NA	38 (71%)	42 (81%)
GN	2 (3%)	NA	28 (54%)
Mac	0	0	NA
No Significance	6 (12%)		

Tryptic Soy Broth (TSB), Gram-negative Broth (GN), MacConkey Broth (Mac).

**Table 3:** Percentage of isolates with significantly different growth in each broth with stool (*n* = 25).

Broth	TSB	GN	Mac
TSB	NA	23 (92%)	25 (100%)
GN	0	NA	8 (32%)
Mac	0	0	NA
No Significance	0		

Tryptic Soy Broth (TSB), Gram-negative Broth (GN), MacConkey Broth (Mac).

## Data Availability

Not applicable.

## Supplementary Materials

The following are available online at https://www.mdpi.com/2076-2607/9/3/503/s1, Table S1: Bacterial Isolate Characteristics.
