# Supplementary material for: Comparison of Common Enrichment Broths Used in Diagnostic Laboratories for Shiga Toxin—Producing Escherichia coli
Source: Microorganisms. 2021 Feb 27;9(3):503. doi: 10.3390/microorganisms9030503 (PMC7997271; doi:10.3390/microorganisms9030503)
Supplement: Supplementary file 1 [file microorganisms-09-00503-s001.pdf]

Supplementary Table S1. Bacterial Isolate Characteristics

Bacterial Isolate	Serotype	*stx* status
1	O45	1
2	O45	1
3*	O45	1
4*	O26	1
5*	O26	2
6	O26	1
7*	O26	1
8	O26	1
9	O26	2
10	O103	1
11	O103	1
12	O103	1
13*	O103	1
14	O103	1
15*	O103	1
16	O111	1 and 2
17*	O111	1 and 2
18	O111	1
19	O111	1 and 2
20*	O111	1
21	O121	2
22*	O121	2
23	O121	2
24	O121	2
25*	O121	2
26	O121	2
27	O145	2
28	O145	2
29*	O145	2
30*	O145	1
31	O145	1
32	O118	1
33	O118	1
34*	O118	1
35*	O118	1
36*	O71	1
37*	O71	1
38	O71	1
39*	O146	1 and 2
40	O113	2

41*	O85	2
42*	O38	1 and 2
43	O91	1 and 2
44	O91	2
45*	O22	1
46	O157	2
47	O157	1 and 2
48*	O157	1 and 2
49*	O157	2
50*	O157	2
51*	O5	1
52*	O Rough	1
* Indicates which isolates were used for the stool spiking experiments		
